# The utility of clinical care pathways in determining perinatal outcomes for women with one previous caesarean section; a retrospective service evaluation

**DOI:** 10.1186/1471-2393-10-62

**Published:** 2010-10-14

**Authors:** Sikolia Z Wanyonyi, Robinson N Karuga

**Affiliations:** 1 Department of Obstetrics and Gynaecology, Aga Khan University Hospital, 3rd Parklands Avenue, Nairobi, Kenya; 2 Research support unit, Aga Khan University Hospital, 3rd Parklands Avenue, Nairobi, Kenya

## Abstract

**Background:**

The rising rates of primary caesarean section have resulted in a larger obstetric population with scarred uteri. Subsequent pregnancies in these women are risk-prone and may complicate. Besides ensuring standardised management, care pathways could be used to evaluate for perinatal outcomes in these high risk pregnancies. We aim to demonstrate the use of a care pathway for vaginal birth after caesarean section as a service evaluation tool to determine perinatal outcomes.

**Methods:**

A retrospective service evaluation by review of delivery case notes and records was undertaken at the Aga Khan University Hospital, Nairobi, Kenya between January 2008 and December 2009

Women with ≥2 previous caesarean sections, previous classical caesarean section, multiple gestation, breech presentation, severe pre-eclampsia, transverse lie, placenta praevia, conditions requiring induction of labour and incomplete records were excluded. Outcome measures included the proportion of eligible women who opted for test of scar (ToS), success rate of vaginal birth after caesarean section (VBAC); proportion on women opting for elective repeat caesarean section (ERCS) and their perinatal outcomes.

**Results:**

A total of 215 women with one previous caesarean section were followed up using a standard care pathway. The median parity (minimum-maximum) was 1.0[1-4]. The other demographic characteristics were comparable. Only 44.6% of eligible mothers opted to have a ToS. The success rate for VBAC was 49.4% with the commonest (31.8%) reason for failure being protracted active phase of labour. Maternal morbidity was comparable for the failed and successful VBAC group. The incidence of hemorrhage was 2.3% and 4.4% for the successful and failed VBAC groups respectively. The proportion of babies with acidotic arterial PH (< 7.10) was 3.1% and 22.2% among the successful and failed VBAC groups respectively. No perinatal mortality was reported.

**Conclusions:**

Besides ensuring standardised management, care pathways could be objective audit and service evaluation tools for determining perinatal outcomes.

## Background

The creation of care pathways has become a popular response to concerns regarding the implementation of evidence-based practice. Care pathways could be a methodology for the mutual decision-making and organization of care for a well-defined group of patients during a well-defined period with the aim to enhance the quality of care by improving patient outcomes, promoting patient safety, increasing patient satisfaction, and optimizing the use of resources [1]. They map the whole journey for a typical patient with a specific diagnosis and include the contribution of the multidisciplinary team. Documentation forms part or all of records of the patients' care. Care pathways could also be handy audit tools for clinical practice [2]. Many obstetric conditions are best managed in a standard way using well designed protocols. These tools could be used to enhance perinatal outcomes in most conditions. This is evident from results on the use of care pathways in critical care, surgery and anaesthesia [3-6].

There has been a persistent concern in obstetrics about the increasing rate of primary cesarean section. This is not restricted by geographical location. Rates higher than those recommended by the World Health Organisation (WHO) have been reported in most parts of the world including developing nations [7,8]. The increased rate of caesarean section inevitably translates to a higher proportion of women with scarred uteri. This poses a challenge to the management of subsequent pregnancies as they become more risky than non scarred uteri and are prone to complications especially where vaginal birth after caesarean section (VBAC) is practised. There has been concerns about the safety and appropriateness of VBAC with reports of poor perinatal outcomes associated with the test of scar (ToS). Reports emanating from well designed studies have led to doubt on the safety of the practice of VBAC with subsequent diminishing acceptance rates [9-12]. The concerns about perinatal outcomes coupled with litigation pressures have also led to the introduction of stringent measures in most developed countries based on the available evidence [13,14]. The practice of VBAC has however persisted in most countries in sub-Saharan Africa despite lack of clear evidence based guidelines like the ones used in the industrialized nations [15-18]. Absence of such guidelines could compromise both maternal and fetal safety. The use of institutional protocol-based care could reduce the incidence of such adverse events in mothers with previous cesarean section if the recommended interventions are implemented [19]

In this study we use evidence based clinical care pathway as a service evaluation tool to determine perinatal outcomes among women with one previous caesarean section in a tertiary teaching hospital in a developing country. Our main aim was to establish whether this tool could be used to assess the perinatal outcomes of women undergoing a test of scar after one previous caesarean section.

## Methods

The study was conducted at the Aga Khan University Hospital, Nairobi. Being a non-experimental service evaluation study no ethical approval was required as per University Hospital's research committee regulation. However the necessary departmental and institutional approvals were obtained.

Aga Khan University Hospital is a private tertiary teaching hospital in Kenya. It is a 254 bedded general facility with over 2,500 deliveries each year. The hospital has a current caesarean section rate of 25-30% with one previous caesarean section accounting for 38% of all elective caesarean sections deliveries. Over the years there have been efforts to reduce this apparently high rate and one such initiative has been encouraging mothers with one previous caesarean section due to a non recurring indication to undergo a ToS. Other initiatives include use of fetal blood scalp sampling to determine fetal wellbeing for suspicious and abnormal cardiotocographic tracings, use of instrumental delivery for second stage disorders and patient education on the benefits of vaginal delivery.

Prior to September 2007, minimal eligibility criteria for ToS at our unit included willingness of the mother to undergo VBAC, non-recurrent indication for the previous cesarean section, and a satisfactory true conjugate determined by computed tomography (CT)/X-ray or clinical pelvimetry. However, like most units elsewhere, we no longer perform pelvimetry as it is widely considered that the fetal head is the best pelvimeter and any other test could be misleading.

These criteria were revised and based on the current evidence a structured clinical pathway was developed for use by all health care providers (see additional file 1). Care is initiated from 20 weeks gestation after confirming fetal normality. The indication and details of previous caesarean section are fully discussed, the relative merits and disadvantages of ERCS and ToS are explored and a mode of delivery is chosen. The women are then assessed for eligibility for VBAC and information leaflets are issued. The details of all the discussions are recorded in the case notes and the care pathway checklists completed appropriately. Further discussions on VBAC are carried out in subsequent visits. The women are again seen at 36 weeks to confirm suitability and where indicated a repeat ultrasound is carried out to confirm placental location. A final decision on the mode of delivery is confirmed at this visit. Should the woman choose to have a ToS then the care pathway is continued till delivery. Completeness for documentation is confirmed before the woman is discharged. Those who choose to undergo an ERCS have their surgery planned at 39 weeks gestation as per the institutional protocol.

We retrieved the records of women who had one previous caesarean delivery and who were delivered between January 2008 and December 2009. Demographic, prenatal and intrapartum data were extracted. Women with two or more previous caesarean deliveries, a classical uterine incision, a history of multiple pregnancies, breech presentation, severe preeclampsia, transverse lie, placenta praevia or suspected macrosomia were excluded.

A retrospective review of records was undertaken to evaluate perinatal outcomes of women with one previous scar based on the care pathway checklists for the period. All women with one previous caesarean section booked for antenatal care and delivery at the Aga Khan University Hospital were included in the study. Incomplete records or inappropriately filled care pathways were excluded from the final analysis. Patients presenting in labour with a previous caesarean section having been booked and followed up elsewhere were also excluded from the final review.

The demographic characteristics and relevant history were captured on a structured data collecting form. The indications and details of the previous caesarean section were also recorded. The perinatal outcomes and all relevant medical and obstetric events leading to delivery were recorded as were the maternal and neonatal complications related to the mode of delivery.

The outcome measures were the proportion of eligible women who opted to have a ToS, the VBAC success rate, peripartum complications and completion rates for the care pathways.

Data were managed using Microsoft Excel^® ^spreadsheets and analysed using SPSS^® ^version 15.0. Descriptive statistics were used. Comparisons between groups were expressed as either absolute percentages or by means and medians. Interquartile range (IQR) and standard deviation (SD) were used as measures of spread.

## Results

A total of 278 women with one previous caesarean section were booked at the prenatal clinic and followed up till delivery. Only 215 (77%) had up to date clinical care pathways for VBAC (see Additional file 1) and were included in the final analysis. The median parity (minimum-maximum) was 1.0[1-4].

Of these 187 (87%) were evaluated and found suitable for VBAC, having met the set eligibility criteria.

**Figure 1:**
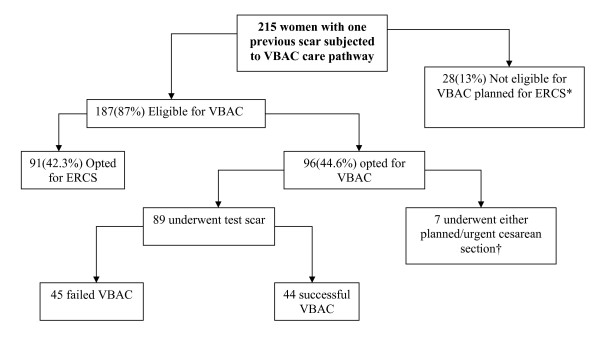
**Management plan for women with one previous scar using the care pathway**. * Postdatism; 11, Absolute CPD; 3, Medical complications requiring induction of labour (hypertensive disease; 3, Gestational diabetes; 2, PMTCT for HIV; 3), Persistent breech presentation; 4, Suspected macrosomia; 1, Previous myomectomy; 1. †Prelabour rupture of membranes requiring induction;4, Gave up test of scar after having initially agreed for VBAC; 1, Undiagnosed breech in labour; 1, Compound presentation in early labour; 1.

Figure 1 below illustrates the management of women with one previous scar using the VBAC care pathway for the period under review

The demographic characteristics of the patients in the two main groups were compared as presented in table 1 below.

**Table 1:** Characteristics of women

Characteristic	Test of Scar Mean (SD)	ERCS Mean(SD)
Age (years)	31.04 (4.1)	31.72 (3.7)

BMI	28.3 (4.3)	29.5(4.8)

Parity; median(min-max)	1(1-4)	1(1-3)
1 > 1	86.5% 13.5%	86.2% 13.8%

Interval from last pregnancy (months)	42.7 (18.2-62.5)	44.6(18.4-64.2)

Information leaflet given (documented) %	100	100

The indications for the previous caesarean section among the 89 women who underwent a trial of labour (VBAC) were compared based on the outcome of labour (i.e. whether VBAC was successful or not) and are listed in table 2;

**Table 2:** Indication for previous cesarean section based on outcome

	Successful VBAC		Failed VBAC	
	**Frequency**	**Percent**	**Frequency**	**Percent**

Fetal distress	25	56.8	14	31.1

Labour dystocia (1^st ^stage)	1	2.3	10	22.2

Malpresentation	5	11.4	6	13.3

Failed induction of labour	4	9.1	4	8.9

Severe Pre-eclampsia	4	9.1	-	-

Malposition	2	4.5	5	11.1

Cephalopelvic disproportion	1	2.3	4	8.9

Others	2	4.5	2	4.4

**Total**	**44**	**100.0**	**45**	**100.0**

The demographic and labour characteristics of women with failed and successful VBAC were also compared table 3.

**Table 3:** A comparison of women's characteristics according to labour outcome

Characteristic	Successful VBAC;	Failed VBAC;
Age; *median (min-max)*	30(24-40)	31(21-41)

BMI	27.3(21.5-35.6)	27.8(21.5-39.5)

Parity	1(1-3)	1(1-2)
1 > 1	87.2% 12.8%	86.4% 13.6%

Interval between cesarean and LMP, months	41.9(17.2-62.1)	43.6(18.3-63.2)

No. of previous successful VBAC	4(1-4)	4(1-4)

Cervical dilation at presentation	4(1-10)	3(1-10)

Station at presentation	*frequency (n = 44)*	*frequency(n = 45)*
Less than -2 -1 0 1 and above	5 12 22 5	7 15 20 3

Duration of 1^st ^stage(hrs); *mean (SD)*	6.6(2.5)	6.9(3.8)

Women who had a successful VBAC had a mean duration of second stage of 22.5 minutes (SD 14.1) and 3^rd ^stage duration of 4.1 (SD 3.8). Two women had a vacuum-assisted delivery due to delayed progress and fetal distress in second stage of labour.

**Figure 2:**
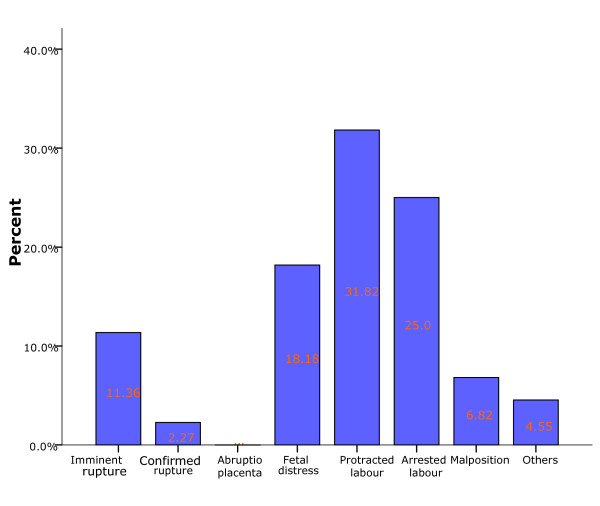
**Reasons for failed VBAC**.

The main reasons for failed VBAC are shown in Figure 2;

The median time taken from the diagnosis of failed VBAC to delivery of the baby was 30.0 minutes (IQR; 10-90).

### Delivery complications

There was only one case (2.3%) of post partum hemorrhage (PPH) due to uterine atony among the women who had a successful VBAC. This was managed with uterotonics in the delivery suite with a good outcome. The other 43 women did not have any delivery related complications.

Maternal complications among the failed VBAC group included severe PPH; 2(4.4%), intraoperative bladder injury;1 (2.2%). These outcomes compared with those for women who underwent an ERCS were severe PPH 2 (2.2%), intraoperative bladder injury 1(1.1%). One woman (2.2%) undergoing a trial of labor had a uterine rupture diagnosed intrapartum by sudden cessation of contractions and a fetal bradycardia. An emergency operation was undertaken with uterine repair. Only one woman (1.1%) scheduled for an ERCS with placenta praevia had a hysterectomy due to placenta accreta. The decision was undertaken intraoperative after a failed attempt of compression sutures to control the bleeding. There were two cases (4.4%) of uterine dehiscence among the ERCS group and none among the failed ToS group.

The mean (SD) post delivery hemoglobin was 11.2 g/dl (1.5) and 10.9 g/dl (1.5) for the successful VBAC and failed VBAC groups respectively.

The neonatal outcomes and complications for the different groups are presented in table 4 and 5

**Table 4:** Neonatal outcomes

	Successful VBAC*	Failed VBAC*
Birth Weight (mean, SD)	3151.6 (402.3)	3297.0 (461.8)

APGAR score n (%) < 7 at 5 minutes	1(2.3%)	0(0%)

Arterial PH n(%) < 7.10	1(3.1%)	8(22.2)

Venous PH % n(%) < 7.15	0(0%)	7(19.4)

*Umbilical cord gas analysis was performed in 32 (72.7%) of babies born following a successful VBAC and 36(81.8%) of those born after failed VBAC.

**Table 5:** Neonatal complications

	Successful VBAC	Failed VBAC
Birth Asphyxia	0	1 (2.8%)

NEC*	0	1(2.8%)

Sepsis	1 (2.3%)	2 (5.6%)

Respiratory distress syndrome	0	2(5.6%)

*Necrotising Enterocolitis

## Discussion

In this study the use of clinical care pathways standardised the practice of VBAC and enabled objective comparisons and perinatal outcomes to be determined conveniently. The overall perinatal outcomes were found to be similar in women with one previous scar regardless of the mode of delivery, even though the proportion of babies with acidotic arterial PH was higher after failed than successful VBAC. More women (44.6%) opted for an ERCS than a ToS (42.3%) and this raised concern since higher acceptance rates have been reported elsewhere [20]. The success rate for ToS was 49.4%. Most of the women were para 1 gravida 2 (87%) and so the influence of previous vaginal delivery after cesarean section could not be reliably determined. All the women who opted for ToS presented in spontaneous labour, however, two mothers had their labour augmented with oxytocin with favorable outcomes. Two mothers also had assisted vacuum delivery with good perinatal outcomes. Our unit is consultant led and decisions to augment labour or perform instrumental deliveries have to be approved by the consultant in charge as stipulated in the care pathway.

A recent systematic review by Rossi et al reported a 73% success rate for ToS and found the incidence of maternal morbidity to be similar for women choosing either ToS or ERCS [21]. A previous study had however found less favorable perinatal outcomes among women who had a ToS [10]. We focused on the outcome of those mothers who attempted a ToS. In our opinion comparing this group with those who opted for an ERCS would have been imprudent considering the two groups were exposed to different risk profiles [13,22]. A short inter-pregnancy interval [23], birth weight [24], maternal diabetes [25], obesity and excessive weight gain [26,27] and lesser degree of cervical dilatation at admission [28] have all been found to influence the success of ToS. This was not tested for in our study as it was not our primary objective. The incidences of poor maternal outcomes e.g. hysterectomy, blood transfusion, uterine rupture, uterine dehiscence, visceral injury and post partum hemorrhage were negligible. Any comparisons arising from these would have been spurious.

The neonatal outcomes, varied according to the mode of delivery, with the proportion of babies born with arterial PH less than 7.10 being higher after failed compared to successful VBAC; 22.2% and 3.1% respectively. These observations compared to findings by Landon et al who reported more acidotic arterial cord PH for the babies born of mothers with failed ToS [10]. Despite these differences in the immediate peripartum period, subsequent neonatal morbidity was comparable regardless of the mode of delivery.

This study is descriptive and data presented do not demonstrate a causal-association relationship. The study does not compare outcomes against a control or a set standard and therefore these conclusions should be interpreted with caution. The study however does demonstrate the use of a clinical pathway as a convenient service evaluation tool in obstetric care. Being a retrospective study we did not evaluate for maternal satisfaction with ToS but identified this as an important inclusion in the patient care pathway during the next revision. Some of the major limitations encountered in our unit included lack of previous operation details as mothers who changed their care providers did not have their previous delivery records. This makes a decision on whether to offer ToS difficult. At institutional level, there were cases of incomplete care pathways and failure to consistently perform cord blood analysis. However, we attempted to objectively compare outcomes among women undergoing a ToS using existing tools to establish whether they were suitable for audit, having put in place mechanisms to ensure maternal and fetal safety. Our study stratified the women into three groups i.e. ERCS, failed VBAC, successful VBAC and made comparisons for the latter two, who in our opinion had similar risk exposure. The women who underwent an ERCS but had hoped to have a ToS or who were found unsuitable for ToS were also accounted for in our study. The information generated clearly demonstrates how safely ToS could be undertaken in a resource limited setting, with careful patient selection, teamwork and appropriate guidelines at all levels of care.

It is evident that acceptance rates for ToS were low and the VBAC success rate was also lower than the commonly quoted figures of 72-76% [14,21]. However, the fact that the perinatal outcomes were comparable regardless of the mode of delivery points to the fact that the practice of VBAC as offered in our institution is safe and efforts should be made to convince more suitable mothers to undergo the process. Factors contributing to lower acceptance rates also need to be determined. The level of satisfaction and factors associated with both successful VBAC and optimal outcomes could be included in revised care pathways.

## Conclusions

Despite its limitations our study was able to demonstrate how a well designed clinical pathway could be used to evaluate service delivery outcomes. Using this tool we were able to determine the perinatal outcomes of VBAC as practiced in our unit. We therefore recommend the use of care pathways for the implementation of evidence based medicine and optimizing prenatal outcomes in obstetric practice. Furthermore, care pathways could also be objective audit and service evaluation tools.

## Competing interests

The authors declare that they have no competing interests.

## Authors' contributions

SZW conceived the study, participated in its design, formulated the study protocol and participated in data collection, literature review and drafting of the manuscript. RNK participated in the design of the study, data collection and statistical analysis. All authors read and approved the final manuscript.

## Funding

None

## Pre-publication history

The pre-publication history for this paper can be accessed here:

http://www.biomedcentral.com/1471-2393/10/62/prepub

## Supplementary Material

Additional file 1 **Appendix: Vaginal birth after previous Caesarean section: care pathway**. The clinical care pathway used for the management of vaginal birth after caesarean section (VBAC) at the Aga Khan University Hospital. Click here for file
