# Supplementary material for: The utility of clinical care pathways in determining perinatal outcomes for women with one previous caesarean section; a retrospective service evaluation
Source: BMC Pregnancy Childbirth. 2010 Oct 14;10:62. doi: 10.1186/1471-2393-10-62 (PMC2964563; doi:10.1186/1471-2393-10-62)
Supplement: Additional file 1 — Appendix: Vaginal birth after previous Caesarean section: care pathway. The clinical care pathway used for the management of vaginal birth after caesarean section (VBAC) at the Aga Khan University Hospital. [file 1471-2393-10-62-S1.DOC]

Patients Name:___________________

Reg. No: ________________________

**Appendix**

**Vaginal birth after previous Caesarean section: care pathway**

| **Antenatal clinic** | **Signed and dated** |
| --- | --- |
| Medical review (after ANP and 20 week scan): assess prospects for safe VBAC. Consider previous delivery setting known, no overwhelming contraindications |  |
| Verbal information to the patient: quote 70% chance of vaginal delivery if labour onset is spontaneous. 1:200 uterine scar dehiscence risk after one CS, 1:50 after two. Advise on need for hospital based care when labouring after previous CS. |  |
| Provide leaflet about VBAC and offer vaginal delivery package if not already offered. |  |
| From 36 weeks: assess prospects for safe VBAC. Absence of contraindications eg breech, placenta praevia, anticipated very large baby, pre-eclampsia (mild non-proteinuric hypertension with normal biochemistry may not be a contraindication) |  |
| From 36 weeks: advise on the need to come to hospital early if labour is suspected, eg even mild contractions, ‘show’ or PROM. Advise the patient to make specific plans for transport to hospital especially at night. |  |
| Post term: advise CS at 41 weeks if undelivered. (Consultant decision to offer induction only if very favourable, eg ARM feasible. Vagiprost is contraindicated). |  |
| **Admission in suspected labour** | |
| Rapid assessment (within 15 minutes of arrival) of general condition and auscultation of fetal heart. Commence admission CTG. |  |
| Resident informs on call obstetric registrar of the admission and makes/ checks the following arrangements (tick each and sign):   - current status of theatre availability - recent haemoglobin is known - group and crossmatch one unit of blood - neonatal unit resident is informed of the case - resident anaesthetist is aware of the admission and comes to review the patient - check that antacid and feeding protocol has been complied with - consent form for emergency CS completed, with advice on page 2 that the procedure will be needed if labour does not progress normally (anaesthetic section can also be completed at this point) |  |
| Midwife in charge confirms adequacy of staffing and informs duty manager if there are any problems |  |
| Midwifery and obstetric staff agree a plan for care and monitoring the patient: eg initial ambulation with intermittent CTG traces in very early/ pre-labour, increasing intensity of monitoring as labour progresses. Plan for analgesia. Complete partogram as for routine care. | (Midwife and doctor sign) |
| Inform consultant on duty of any deviation from normal progress including abnormal CTG, clinical suspicion of dehiscence, arrest in labour, abnormal bleeding. Do not commence syntocinon without consultant authorization. | (Resident/ registrar sign and add name of consultant) |
| Update anaesthetist, theatres and neonatal unit about progress in late first stage of labour, or lack of progress whenever evident |  |
| **Second stage** | |
| Continue electronic monitoring as much as possible |  |
| Assess non advance, consider intervention after 30 minutes |  |
| Operative vaginal delivery may be appropriate if the conventional criteria are fulfilled. Consider a trial of vaginal delivery in theatre if any adverse features are present (involve registrar/ on call consultant in this decision) |  |
| **Emergency CS in labour** | |
| Inform on-call consultant | (Resident/ registrar sign and add name of consultant) |
| Continue auscultation during transfer to theatre and anaesthetic preparation where possible, eg with sonicaid |  |
| Document time of decision to proceed to CS here:  Time of delivery here: |  |
| **Post delivery (vaginal or CS)** | |
| Obtain cord gases, provide the results to the paediatrician immediately and document the results clearly in the notes.. Check that CTG traces are filed in the case notes. |  |
